# Efficacy of early chick nutrition with *Aloe vera* and *Azadirachta indica* on gut health and histomorphometry in chicks

**DOI:** 10.14202/vetworld.2017.569-573

**Published:** 2017-06-01

**Authors:** Tamilvanan Sujatha, Sivasankar Abhinaya, Jai Sunder, Marudhai Thangapandian, Anandamoy Kundu

**Affiliations:** Division of Animal Science, ICAR-Central Island Agricultural Research Institute, Port Blair - 744 105, Andaman and Nicobar Islands, India

**Keywords:** *Aloe vera*, *Azadirachta indica*, chicks, early chick feeding, gut health, histomorphometry

## Abstract

**Aim:**

This study was conducted with an aim of studying the efficacy of water supplements of *Aloe vera* and *Azadirachta indica* (neem) during pre-starter age (0-2 weeks) on gut health and histomorphometry in Vanaraja chicks.

**Materials and Methods:**

A total of 192 day old Vanaraja chicks were randomly assigned to one of four herbal water treatments throughout the experimental pre-starter stage (0-2 weeks) in a completely randomized design. Each treatment was given four replicates consisting of 12 chicks per replicate. Water treatments comprised T1: Control with regular antibiotic supplement, T2: 3 ml *Aloe* juice per chick per day, T3: 3 ml neem extract per chick per day, T4: 1.5 ml *Aloe* and 1.5 ml neem per chick per day. Gut culture was done for *Escherichia coli* and *Lactobacillus* sps. and gut histomorphometry in 24 gut samples at 14 days of age.

**Results:**

This study revealed that supplementation of *A. vera* and neem in water significantly (p<0.05) reduced and increased the number of gut *E. coli* and *Lactobacillus* sps. Colonies, respectively, as compared to that of control groups; Villi was significantly (p<0.05) taller and broader on 14 days of age across the jejunum of chicks fed with neem supplementation as compared to that of control chicks. Significantly lower crypt depth (p<0.05) was observed in the duodenum of *Aloe* supplementation. Villus height: Crypt depth ratio of duodenum and jejunum was significantly (p<0.05) increased neem and *Aloe* supplementation in chicks as compared to their combination and control.

**Conclusion:**

Immediate post hatch supplementation of Aloe juice and neem extract in chicks improved the development and health of their gut.

## Introduction

Early chick nutrition, commonly termed as pre-starter feeding helps in utilization of yolk sac for optimal immunity and gut development and to enhance its function. Early chick feeding has a great potential to trigger the gut and its morphological development for improved performance and better feed conversion ratio [1,2]. Hence, studies on the effect of early chick nutrition on gut health and morphology are having great importance in poultry. Since the start of organized poultry industry, antimicrobials have been used as feed and water supplement in poultry feed to enhance the gut development and growth performance. However, due to emergence of multiple drug resistant bacteria [3] by the use of antibiotics at sub-therapeutic level, pre and probiotics, herbs, spices and various medicinal plant extracts are being given more attention as possible antibiotic growth promoter replacement [4].

Medicinal plants have an indispensable source of medicine for poultry production systems since ancient times. A significant number of farmers are still judiciously using herbal remedies in the management of rural poultry in spite of modern veterinary treatment. As per the estimation of WHO, even today 80% of people still rely on medicinal plants [5] for their livestock treatment. Studies on the use of phytogenic feed additives as growth promoters and immune enhancers in broiler nutrition are numerous. *Aloe vera* has a rich source for many chemical compounds and plays various roles in animal system. Similarly, *Azadirachta indica* (neem) leaves have vast properties such as as immune modulatory, anti-inflammatory, antihyperglycemic, antimalarial, antifungal, antibacterial, antiviral, antioxidant, antimutagenic, and anticarcinogenic [6-8]. Neem plays an important role enhancing growth owing to antibacterial and hepatoprotective properties [7]. However, work on effect of early chick nutrition with *Aloe* and neem juice on gut histomorphology is limited.

The morphology of intestinal villi and crypts has been associated in chickens with intestinal function and growth. Adverse changes in the content of the digesta, such as high population of pathogenic bacteria, parasites or substances, could lead to changes in the surface of intestinal mucosa, because of their close proximity. Hence, the aim of this work is to study the efficacy of water supplementation of *A. vera* and *A. indica* during pre-starter age (0-2 weeks) on gut histomorphometry of Vanaraja chicks.

## Materials and Methods

### Ethical approval

The experiment was conducted after the permission of Institutional Animal Ethics Committee of ICAR-Central Island Agricultural Research Institute, Port Blair.

### Sample preparation and experimental design

*A. vera* and *A. indica* (neem) were freshly collected for the study. *A. vera* juice was prepared by grinding the pulp without water, filtering and mixing with water at 1:1 ratio; 200 g of neem leaves in 1 L of water was kept in shaking water bath for overnight for extraction and was filtered in the morning. The pre-starter feed was prepared with 20% crude protein and 2800 kcal ME/kg as per BIS (2007) recommendation and fed *ad libitum* to the experimental chicks. A total of 192-day-old Vanaraja chicks were randomly assigned to one of four herbal water treatments throughout the experimental pre-starter stage (0-2 weeks) in a completely randomized design. Each treatment was given four replicates consisting of 12 chicks per replicate. Water treatments comprised T1: Control with regular antibiotic supplement, T2: 3 ml *Aloe* juice per chick per day, T3: 3 ml neem extract per chick per day, T4: 1.5 ml *Aloe* juice and 1.5 ml neem extract per chick per day.

### Data collection

Tissue samples from duodenum, jejunum and ileum and internal contents of intestine and ceca were collected from six Vanaraja birds by slaughtering, from each treatment by humane method at 14^th^ day of age. The gut contents were cultured for *Escherichia coli* and *Lactobacillus* sps. in nutrient broth and MacConkey Rogosa (MRS) broth, respectively, and identified using specific respective medias of eosine methylene blue and MRS agar. The microbial counts were determined as colony forming units per gram of samples (n=6 per treatment). The histomorphological study of tissue samples (n=6 per treatment and 6 fields per sample) was carried out according to the method described by Bancroft and Marilyn [9]. The data generated out of observations were subjected to statistical analysis as per Petrie and Watson [10]. The significance of the difference among the groups was determined by Duncan’s multiple range tests [11].

## Results

### Effect of *Aloe* and neem water additives on gut microflora

Present experiment showed that supplementation of *A. vera* and neem in water significantly (p<0.05) reduced and increased the number of gut *E. coli* and *Lactobacillus* sps. colonies, respectively, as compared to the control (Table-1).

**Table-1:** Effect *Aloe* and neem water additives on gut microbes in Vanaraja chicks.

Treatments	Intestine		Cecum	
	
	*Lactobacillus* sps.	*E. coli*	*Lactobacillus* sps.	*E. coli*
Control	4.06^c^×10^10^	39.6^a^×10^10^	14.9^c^×10^10^	93.5^a^×10^10^
*Aloe*	9.36^a^×10^10^	4.07^b^×10^10^	31.8^ab^×10^10^	38.6^b^×10^10^
Neem	10.36^a^×10^10^	0.72^c^×10^10^	47^b^×10^10^	21.65^c^×10^10^
*Aloe*+neem	5.38^b^×10^10^	2.6^b^×10^10^	60^a^×10^10^	14.68^d^×10^10^

E. coli=Escherichia coli

### Effect of *Aloe* and neem water additives on histomorphological parameters of the gut villi in Vanaraja chicks

**Plate-1:**
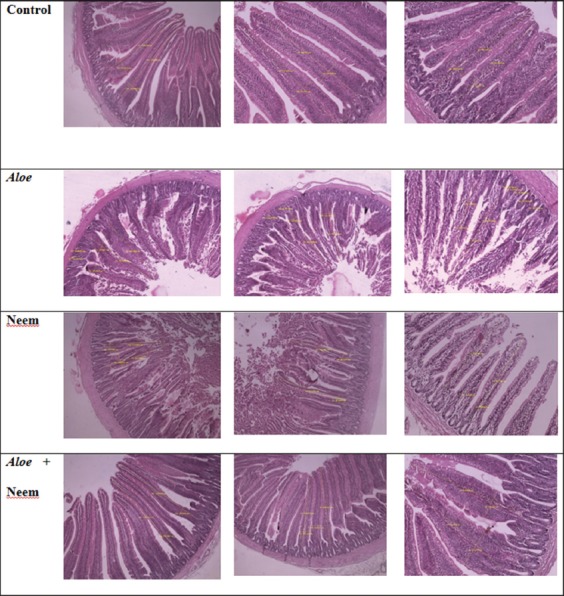
Effect *Aloe* and neem water additives on gut histomorphometry in Vanaraja chicks.

Villus height, width, and depth in the different segments of the small intestine of vanaraja chicks fed with *Aloe* and neem is shown in Table-2 and Plate-1. Histomorphometry of villi were significantly influenced by herbal supplementation. The significantly higher (p<0.05) villi was seen in jejunum and illeum when neem was supplemented and that was statistically comparable with *Aloe* supplementation in duodenum and with *Aloe* plus neem supplementation in illeum, whereas there was significantly lower villi seen when chicks fed without any supplementation. However, there was no significant difference in villi height of duodenum, jejunum and ileum with *Aloe* plus neem supplementation as compared to the control.

**Table-2:** Effect *Aloe* and neem water additives on gut histomorphometry in Vanaraja chicks.

Treatments	Villi height (µm)			Villi width (µm)			Crypt depth (µm)			Height: crypt ratio of villi		
			
	Duodenum	Jejunum	Ileum	Duodenum^NS^	Jejunum*	Ileum^NS^	Duodenum*	Jejunum^NS^	Ileum*	Duodenum*	Jejunum*	Ileum^NS^
Control (n=6)	858.1±45.8^b^	570.9±10.0^b^	514.5±20.0^b^	110.4±9.2	85.5±12.6^b^	98.8±8.5	136.88±3.6^a^	136.31±16.7	126.40±8.3^a^	6.27±13.6^b^	4.18±3.6^b^	4.07±17.3
*Aloe* (n=6)	1271.0±27.6^a^	808.2±34.3^a^	597.9±18.8^ab^	147.7±20.2	111.7±6.2^a^	107.0±9.8	86.72±15.7^b^	125.39±7.07	112.57±2.0^ab^	14.66±8.9^a^	6.44±8.3^a^	5.31±11.1
Neem (n=6)	1251.0±14.5^a^	902.8±46.9^a^	664.7±16.6^a^	170.0±18.4	126.9±7.5^a^	100.3±10.6	91.07±9.3^ab^	120.55±1.0	96.22±4.4^b^	13.74±15.7^a^	7.48±10.7^a^	6.91±18.5
*Aloe*+neem (n=6)	958.2±75.5^b^	657.3±34.9^b^	538.2±27.4^b^	172.2±21.8	115.2±6.3^a^	137.3±15.9	124.55±12.9^ab^	140.56±9.7	92.34±8.8^b^	7.69±20.6^b^	4.67±23.9^b^	5.83±21.7

Mean values having same superscripts within column do not differ significantly.

* Significant, **Highly significant. NS=Non significant

With regard to villi width, significantly (p<0.05) broader width was recorded in jejunum at 14^th^ day of supplementation with neem as compared to control and was statistically comparable with other herbal supplementation among which neem supplementation had induced significantly broadest villi. There were no significant differences in villi width of duodenum and illium between herbal supplementation and control.

Significantly (p<0.05) lower crypt depth was seen with neem and *Aloe* and *Aloe* plus neem supplementation as compared to control. Significantly lower crypt depth (p<0.05) was observed in the duodenum when the *Aloe* was supplemented as compared to neem and *Aloe* plus neem supplementation. Water supplementation of *Aloe*, neem and their combination did not result in significant differences in the crypt depth of jejunum segment. Villus height: Crypt depth ratio of duodenum and jejunum was significantly (p<0.05) increased at 14 days of neem and *Aloe* supplementation in chicks as compared to their combination and control. Small lumen and epithelium (Plate-1) were observed in the intestinal glands attached to the villi of control chicks. The inter-glandular corrion connects both the lymphatic and capillary network and was loaded with many infiltrate cells. The capillary network showed evidence of hyperplasia and hypertrophy, whereas the chicks from *Aloe* and neem supplementation had villi with intestinal glands of the duodenum having a large lumen and were surrounded by thin interglandular spaces, with the interglandular villi containing collagen fibers, fibroblasts and leukocytes infiltrate. The presence of the capillary ectasia in the main villi and interglandular villi in the present images of duodenum suggest that the angiogenesis process has been stimulated. The capillary network underwent both hyperplasia and hypertrophy and seen to be powerfully stimulated by the lymphoid infiltrate.

## Discussion

### Effect *Aloe* and neem water additives on gut microbes in vanaraja chicks

The microbial population in the gastrointestinal tract of poultry plays an important role in normal digestive processes and health maintenance [12]. This microbial shift might be due to the presence of huge numbers of chemically diverse and biologically active ingredients [13] in neem and acemannan, the polysaccharides in *A. vera* [14]. The results are in agreement with Mohammadmehdi and Jamshid [15] who also reported increased *Lactobacillus* spp. and reduced *E. coli* count through supplementing the feed with *A. vera* gel and Poonam *et al*. [16] as well recorded broad spectrum antibacterial effect with neem leave extract. Our findings are in accordance with reports that medicinal plants as alternatives to antibiotics exhibit the direct or indirect effects on intestinal microflora in poultry. This might be due to establishment of beneficial *Lactobacillus* sps. in that intestine. The final fermentation product of *Lactobacillus* sps. is lactic acid that might has made the gut environment unfavorable for pathogens and modified harmful microbial population.

### Effect *Aloe* and neem water additives on gut histomorphometry in Vanaraja chicks

It was observed that neem and *Aloe* supplementation significantly (p<0.05) improved the height of villi in duodenum, jejunum and ileum (Plate-1 and Table-2) while significant effect of *Aloe* plus neem could not be observed as compared to control. Among *Aloe* and neem supplementation, neem significantly improved both height and width of villi in jejunum. Except jejunum region, neem and *Aloe* supplementation did not significantly influence the width of villi in duodenum and ileum. Crypt depth were (p<0.05) significantly reduced by *Aloe* and neem supplementation in duodenum and ileum except jejunum. Significantly higher villi and lower crypt depth increased their ratio in the duodenum and jejunum of chicks fed with *Aloe* and neem extract. Water supplementation of *Aloe*, neem and its combination did not bring out any significant differences in the villi height and depth ratio of illeum segment. The results of present study are in agreement with the report of Homan *et al*. [17], Kadhim *et al*. [18] that longer villi is essential to animal development because it would result in an increased surface area for absorption of nutrients [19]. Accordingly, the significantly higher ratio of villus height: Crypt depth in the present study indicated that *Aloe* and neem supplementation has made the gut environment free of microbial toxins [20]. Short-chain fatty acids produced by *Lactobacillus* sps. in that intestine are responsible for favorable change in intestinal morphology and might have stimulated the proliferation of epithelial cells of the bowel [21]. In addition, lower crypt depth with *Aloe* and neem supplementation indicated for slow tissue turnover preventing the pathogens from tissue destruction in the gut [20].

## Conclusion and Recommendation

Based on this comprehensive study, it is concluded that immediate post hatch supplementation of extracts of *Aloe* and neem in the water of Vanaraja chicks enhanced the gut health and gut development. The *A. vera* and *A. indica* might be promising replacers to antibiotic growth promoters for organic poultry production.

## Authors’ Contributions

TS contributed in designing of experiment extraction and feeding experiment in poultry. SA did the feeding experiment, monitoring of the experimental birds. JS did the microbial analysis of gut flora. MT did the histopathological analsyis of gut tissues. AK did the analysis and overall monitoring of the experiment and preparation of manuscript. All authors read and approved the final manuscript.
